# Effects of Management Strategies on Non-Beak-Trimmed Laying Hens in Furnished Cages that Were Reared in a Non-Cage System

**DOI:** 10.3390/ani10030399

**Published:** 2020-02-28

**Authors:** Maryse Guinebretière, Amandine Mika, Virginie Michel, Loïc Balaine, Rodolphe Thomas, Alassane Keïta, Françoise Pol

**Affiliations:** 1 Epidemiology, Health and Welfare Unit, French Agency for Food, Environmental and Occupational Health & Safety (ANSES), 22440 Ploufragan, France; loic.balaine@anses.fr (L.B.); rodolphe.thomas@anses.fr (R.T.); alassane.keita@anses.fr (A.K.); francoise.pol@anses.fr (F.P.); 2 Birds and Poultry Biology Research Unit, Centre INRA Val de Loire, Technical Institute for Poultry (ITAVI), 37380 Nouzilly, France; mika@itavi.asso.fr; 3 Direction of Strategy and Programmes, French Agency for Food, Environmental and Occupational Health & Safety (ANSES), 14 rue Pierre et Marie Curie, 94701 Maisons-Alfort, France; virginie.michel@anses.fr

**Keywords:** laying hen, injurious feather pecking, rearing, feather cover, environmental enrichment

## Abstract

**Simple Summary:**

The practice of beak trimming in laying hens limits the negative consequences of injurious pecking, but could be prohibited by future regulations. We addressed in this study the question of how to prevent or minimize injurious pecking and its consequences, especially when beak trimming is not carried out. We found that a combination of different management strategies applied from rearing to laying stages improved to some extent the welfare of non-beak trimmed birds (lower mortality and fear of a novel object, better feather cover up to 61 weeks of age). Fiber supplementation in the feed did not provide any benefit on pecking-related problems. We suggest practical solutions to limit the consequences of injurious pecking where non-beak-trimmed animals are reared in barn system and transferred to furnished cages, while preserving good welfare, health and productivity.

**Abstract:**

Beak trimming in laying hens limits the negative consequences of injurious pecking, but could be prohibited by future regulations. This study assessed a combination of management strategies during the rearing period (objects, perches, music, human presence) and laying period (scratching mats, objects, feed fiber supplementation) to raise non-beak-trimmed animals. The welfare and laying performances of beak-trimmed (T) and non-beak-trimmed (NT) ISA Brown birds were compared between groups with (E) or without (NE) these strategies, with or without fiber supplementation in the diet during laying period. Fiber supplementation did not provide any benefit on pecking-related problems. In comparison with NT-NE birds, NT-E birds had lower mortality, were less fearful of a novel object, and had a better feather cover, without a negative impact on productivity (same laying rate and egg quality). Although this study showed advantages of beak trimming (T birds had higher body weights, laying rates and lower hen mortality than NT birds), it highlighted related problems (increasing pullet mortality, decreasing early weights and increasing beak defects). This study proposes practical solutions to limit the consequences of injurious pecking in non-beak-trimmed animals.

## 1. Introduction

Worldwide, the seven billion laying hens raised every year are mainly housed in cages [1]. In Europe, driven by strong societal sensitivity for animal welfare, the proportion of cage housing systems (furnished ones since 2012) has gradually decreased in recent years. However, 50.4% of laying hens in the European Union (EU) were still housed in furnished cages in 2018 [2]. Furnished cages were initially designed to house relatively small groups of hens (e.g., 20). There is now a trend towards larger group sizes, increasing locomotion possibilities for animals but having implications for some behaviors, for instance the development of injurious pecking (IP). The notion of IP covers a range of behaviors such as aggressive pecking, feather pecking, cannibalism including vent pecking and toe pecking [3]. In this paper, aggressive pecking will be excluded because it has a different cause than the other injurious pecking activities. Injurious pecking is currently the most problematic behavioral issue the poultry industry is facing, with impacts on welfare including health and productivity [4,5]. This problem may occur in all types of housing systems, during rearing of pullets, as well as during the laying period [6,7,8,9], and it is difficult to stop this behavior once it has begun [7]. Injurious pecking is not a new problem: a large amount of research has been carried out to determine the factors influencing IP. However, it is still difficult to systematically prevent and treat because it is a multifactorial problem with a wide range of possible risk factors including genetics, stocking density, group size, floor type, feed, light (intensity, wave length) and others environmental factors [4,10,11,12,13,14,15,16].

Currently, the most effective and reliable method of reducing the damage caused by IP is beak trimming, consisting in most cases of infra-red radiation applied in day-old chicks at commercial hatcheries. The use of this method has become widespread, in a prophylactic manner on all flocks. The procedure appears to be efficient, because IP problems are more common and feather condition is worse in birds with intact beaks, in comparison to beak-trimmed birds [17]. However, IP is also found in beak-trimmed flocks and as a result, beak trimming fails to completely prevent IP-related problems. Even though it is commonly used in egg production, beak trimming is heavily debated for animal welfare reasons, because of the pain or discomfort caused to chicks during and after the trimming procedure [18], despite its improvements in the technique over the years. Therefore, understanding how to manage non-beak-trimmed pullets and hens is important to good ensure animal welfare.

In egg production, day-old chicks are transferred from the hatchery to a rearing farm, and then transferred around 18 weeks of age (WOA) to a variety of layer housing systems. It is recommended that laying hens be reared according to their later housing conditions for optimal welfare and productivity of layers [19]. Generally, producers try to match the type of rearing facility with the type of layer facility, especially for aviary systems, but this is not always possible. In France, one of the leading egg producer in the EU with 1 million tons eggs in 2019 [2], laying hens housed in cages are in 60% of cases housed in barn systems during the rearing period, without any enrichment except litter (personal data—survey used in [14]). Rearing in a relatively spacious environment with access to substrate and other enrichments, followed by transfer to a restrictive environment without any substrate, or with a relatively poor substrate, may cause frustration inducing high reactivity and IP [20].

Several reviews [4,12,21] have been published and management guidelines [10,22,23] developed to help farmers with IP control in free-range, barn and organic systems. Factors that could mitigate this multifactorial problem are multiple. For instance, providing birds with the opportunity to explore and forage reduces the risk of IP [24]. Enriching the environments with suitable litter, various types of plastic toys or string enrichments can reduce IP, thereby improving feather cover [25], with importance of renewing of enrichments [26] to avoid animal boredom and lack of interest, and to train animals to accept novelty. Decreasing animal emotional reactivity and fear is also important [15]. This can be done by giving animals time to adjust or prepare for change, or by providing hens the possibility to escape from feathers peckers thanks to perches or panels [26], or by improving the human-animal relationship [27]. Another aspect influencing IP mentioned in numerous experiments is nutrition (reviewed by [28]). Presenting food in ways that stimulate foraging behavior may increase the time spent eating and decrease the time spent on IP [29]. Finally, the conditions during the rearing period play an important role in the prevention of IP [7,25,30]: with provision of an enriched environment to stimulate foraging behavior [6], or the possibility to perch early to lower the risk of vent pecking during the laying period [31].

Although the literature on the causes of IP and potential solutions is abundant, it is sometimes difficult to apply the findings to the current French situation. Most research on non-beak-trimmed hens focusses on free-range or other cage free birds. In cage housing systems, introducing enrichment and renew it is challenging because of financial costs and time demands, even though it may be more important given the relatively barren environment of a cage [32]. Therefore, simple, robust and inexpensive solutions are required for cages in commercial poultry production. Moreover, research had a strong focus on the laying phase, with less interest on the rearing phase. Finally, experimental studies have often shown the value of the tested strategies in an individual manner. However, the various influencing factors may interact, and the successful transfer of the results of these studies into farming practice is difficult.

The goal of this study was to measure the effect of a combination of several management strategies from rearing to laying stages that have proven to be individually efficient to control IP. The different management strategies were chosen taking into account the potential benefit for the animals, feasibility and cost for farm implementation. The objective was first to decrease reactivity (giving pullets the possibility to escape from feather peckers, providing gradual changes in light to create dusk and dawn phases, providing music), and to decrease animal fear of humans (allowing the animals to anticipate the person’s arrival, improving the human-animal relationship). A second objective was to promote more natural behaviors with an extra environmental enrichment. Concurrently, the enrichment should be feasible, robust and inexpensive for commercial application. Thus, during rearing period, objects were renewed in contrast to the laying period, where objects were deliberately left until the end of the experiment, without being renewed to mimic commercial conditions. A last objective was to increase the time spent eating, by fiber supplementation in the diet during laying period. The hypothesis was that the combination of the different management strategies altogether leads to a decrease of IP and resulting problems related to body status, fear and laying performance. As IP is found in non-beak-trimmed but also in beak-trimmed flocks, comparisons of effectiveness of such management strategies on these two situations were carried out.

## 2. Materials and Methods

### 2.1. Animals and General Husbandry

#### 2.1.1. Rearing Period

ISA Brown day-old chicks (*n* = 4656) were obtained (May 2016) from a commercial supplier: half of them had infra-red beak treatment. Both beak-trimmed (T) and non-beak-trimmed (NT) chicks were randomly allocated to two separate rooms at the experimental facilities of ANSES Ploufragan (France), and reared in groups of 388 in floor pens (surface 30 m^2^) until 17 WOA. In each room, there were six identical pens: three housing T pullets, and three housing NT pullets. In both rooms, pullets had *ad libitum* access to the same commercial rearing feed and water, and wood shavings as litter. They were all reared following standard commercial specifications for temperature, lighting (20 lx), and vaccinations.

The birds in the two separate rooms were subjected to different rearing treatments. In one room—the “enriched” room (E)—a combination of seven management strategies was provided, and these strategies were absent in the other room—the “unenriched” room (NE). These management strategies are grouped in the following paper as “enrichment” even if they did not include exclusively environmental enrichments. They consisted of:

(1) a total of 8 cm/animal of wooden perches: one perch rack in the middle of the pen (H × W: 1.6 m × 2.1 m with 7 perch bars evenly spaced at 40 cm intervals), another at the rear of the pen, close to the back wall (H × W: 1.2 m × 1.9 m with 3 perch bars evenly spaced at 40 cm interval), and 4 additional low perch bars (H × W: 0.2 m × 2 m). They were provided continuously.

(2) the continuous presence of plywood boards (l × L = 1 m × 0.35 m) placed under the two perch racks and maintained vertically as a barrier, allowing animals to retreat.

(3) five objects that were renewed every 15 days, from 2 until 17 WOA, with different object combinations (i.e., 9 object combination changes during the rearing period). The same combination of 5 different objects per pen were present at the same time in each pen. These objects were placed on the ground: PVC pipes (long, elbow or T-shaped around H × W: 10 cm × 25 cm), jerricans (red, white, orange), a sand box (1 m^2^) or suspended items (compact discs, plastic bottles—red, white, orange or transparent filled with colored rings for poultry, strings, paper egg trays and red feeder lids) or items suspended using nets (plastic bales, pecking blocks and straw).

(4) sound enrichment: music playing during lighting hours (radio mixing music and voices) around 30 dB.

(5) an alert system for anyone entering the enriched room: knocking on the door.

(6) lights switched on and off gradually (20 min duration).

(7) additional human passages, several times a week: a human entered the pen, crossed the pen while making regular stops, at the same speed and for the same duration in each pen and came out of the pen.

#### 2.1.2. Laying Period

At 17 WOA, birds (*n* = 4200) were transferred in groups of 60 hens for the laying period up to 73 WOA to furnished cages, according to rearing and beak treatments: E pullets were transferred to E cages, and conversely. The 70 cages were all arranged in a single room at the experimental facilities (ANSES Ploufragan, Ploufragan, France), and distributed into three battery blocks with each three tiers (and aisles in-between). Treatments were allocated equally to each battery and tier.

Each E and NE cage (750 cm^2^/hen) contained a nest (67 cm^2^/hen with an artificial turf mat surrounded by flexible walls) and perches (15 cm/hen). In E cages, a combination of two enrichment strategies was provided, these were absent in the NE cages:

(1) an artificial turf mat for pecking and scratching in an area of the cage free of perches (67 cm^2^/hen),

(2) ten different objects hooked onto the cage facade: small mobile and colored objects (5 to 10 cm) hung by a metallic cable, plastic transparent pots (H × W: 10 × 5 cm) filled with colored rings for poultry and plastic chain links (5 cm).

For the experimental purpose, the housing conditions for NE birds did not fulfil European minimum standards regarding the pecking and scratching area, as no mat was provided in the area free of perches in NE cages.

All hens had *ad libitum* access to water and feed automatically delivered 3 times per day: at 7 am, 3 pm and 8 pm. Two types of diet were provided. As automatic distribution of feed can only be implemented per full battery, one battery (23 cages) received a standard commercial diet (C: control group, 4.01% cellulose), while the others two batteries (47 cages) received a diet with 5.16% cellulose (F: fiber inclusion of Arbocel^®^ in 2% of the total diet, with the same energy formulation as standard diet).

For all cages, the lights (bulbs on 2 levels) were arranged facing the scratching area, opposite the nests (luminosity measured facing the nests: 3 lx and facing the scratching area: 19 lx). Lights were switched on and off gradually (20 min duration) from 6 am to 10 pm from 21 WOA.

### 2.2. Measures

#### 2.2.1. Mortality

During the rearing and laying periods, mortalities were recorded daily. For ethical reasons, birds that were severely cannibalized were culled and recorded as dead.

#### 2.2.2. Body Status: Body Weight, Feather Cover, Skin Damage, Beak Defects

During the rearing period, at days 1, 3, 6, 11, 21, 28, 35, 49, 63, 105 and 119 of age: 50 pullets per pen were weighed in all pens (i.e., 150 birds for each treatment). Pullets were randomly selected in each pen; and for each weighing individuals could be different or identical each time. At 16 WOA, feather cover, beak status and skin damage were assessed on 16 pullets randomly selected in each pen (i.e., 48 pullets per treatment).

During the laying period, at 31, 35, 41 and 61 WOA: feather cover was assessed for all cages on 8 hens randomly selected per cage, and observed in situ from the corridor (without handling) by a single observer. Individuals could be different or identical each time. At 71 WOA: 8 hens randomly selected in each cage were removed from their cages and evaluated ex situ for feather cover, beak status, skin damage and body weight. Numbers of assessed hens were equally distributed between beak and enrichment modalities (140 hens in each B × E treatments). This represented 326 F hens and 234 C hens.

Feather cover was scored on three areas (back, cloaca and tail, without stroking back feathers) using a three-point scale: 0%–20% of the surface area affected by plumage damage (score 0); 20%–50% of the surface area affected by plumage damage and visibly defeathered parts (score 1); 50%–100% of the surface area affected by plumage damage and visibly defeathered parts (score 2). The scores for the three body areas were added together for each hen, with a total score between 0 and six points (6 representing the worst feather degradation).

Beak morphologies were assessed visually to classify them as having a defect: shovel beaks (bottom beak longer than top), split (beaks with a visible crack on the top and/or bottom beak), crossed (bottom on top beaks in two different directions) or not [33].

Skin damage was recorded as no skin lesion over 1 cm long on the whole body area (score 0), or at least one skin lesion over 1 cm long on the whole body area (score 1).

Feather cover, beak status and skin damage were assessed ex situ by three observers firstly trained to do it on 25 birds in common. They assessed the same number of birds in each treatment. The χ^2^ tests showed no difference between the scores they gave: *p* = 0.23 for feather cover score, beak status and skin damage at 16 WOA, and *p* = 0.47, 0.61 and 0.25 for feather cover score, beak status and skin damage at 71 WOA.

#### 2.2.3. Behavioral Tests

Fear or exploratory motivation towards an object and fear or attraction towards an unfamiliar human were assessed using tests in the home environment (following [34] for non-cage systems during the rearing period, and [27] for cage systems during the laying period).

##### Novel Object Test (NOT)

During the rearing period, a novel object test (NOT) was performed at 10 WOA in all pens, between 8 and 11.30 am, by a single observer. The NOT followed the procedures of the Welfare Quality^®^ assessment protocol for poultry [35]. The novel object (NO) consisted of a plastic pipe with tapes of different colors (12 cm long × 2.5 cm diameter). The observer remained immobile for 3 min outside the pen, then counted animals in a defined area of the pen (3 × 1 m, similar between pens) for 2 min by scan sampling at 10-s intervals (total of 12 scans per pen). Then, NO was placed in the middle of the defined area, on the litter by the observer who observed immediately after, from outside the pen, the number of pullets next to the object (distance from an animal) and pecking the object, by scan sampling at 10-s intervals, over a 2-min period (total of 12 scans per pen).

During the laying period, the NOT was performed at 62 WOA between 8 and 11.30 am, by a single observer, in cages from the medium and bottom tiers (upper tier was more difficult to observe directly without disturbing the test) (i.e., total of 46 cages: 11 to 12 cages in each B × E treatment, representing 31 F cages and 15 C cages). After 3 min in the presence of the observer who remained immobile 4 m away from the cage, the observer counted hens with their heads out of the cage façade or immediately behind the façade, by scan sampling at 10-s intervals, over a 2-min period (total of 12 scans per cage). Then, NO was hung on the façade of the cage (same NO as during the rearing period). Immediately thereafter, the observer remained on the corridor 4 m away from the cage, and observed the same parameters than before NO presence, adding the number of hens pecking the object, by scan sampling at 10-s intervals, over a 2-min period (12 scans per cage).

In both tests (during the rearing and laying periods), the numbers were related to the initial situation (average of the 12 scans after the NO placement compared to the 12 scans before) to assess the hens’ reactivity to the NO.

##### Avoidance Distance Test (ADT)

During the rearing period, an avoidance distance test (ADT) was performed at 9, 14 and 15 WOA in all pens by a single observer (hens not familiar with the observer, contrary to the caretakers) between 4 and 6 pm. The observer walked around the pen for 2 min then sat on the floor and waited 3 min for the pullets to become accustomed to the human presence. Then, the observer noted the number of pullets within an arm’s length, tried to gently touch birds and noted the number of pullets touched, the number of pullets pecking at the observer, and the latency of the first peck at the observer.

During the laying period, an ADT was performed at 31, 41 and 53 WOA in cages from the medium and bottom tiers (same sample as the NOT), by a single observer (who was not involved in caretaking), between 4 and 6 pm. The observer walked gently down the corridor along the wall or opposite the battery with one hand held in front of the body, and the other hand held at the side, the body not facing the cage. The observer focused on any hen with its head out of the cage façade, and gently moved forward at the level of the hen (with the hand remaining in front). Then, the observer turned towards the hen and approached her at a speed of one-step per second before withdrawal. A score from 1 to 4 (1: distant (2 m), 4: very close (10 cm)), reflecting the distance between the hand and hen’s feet was noted when the hen put her head back in the cage. If the hen pulled her head in before this step, the observer skipped to a different hen. If more than three hens were skipped, or if no hens had their head out before the test, a score of 0 was given for the cage.

#### 2.2.4. Laying Performance

When the hens were 24, 36, 42, 50, 64, 68 and 72 WOA, the eggs laid on 2 consecutive days were counted per cage for 5 to 7 cages per treatment. Cages were selected randomly at 23 WOA and the same cages were used for the following measures. The eggs from each cage were visually examined to record the number of cracked and dirty eggs. The normal eggs produced per cage (neither dirty nor cracked) were weighed to determine the mean egg weight. Hen-day egg production (eggs laid per cage × 100/number of hens housed per cage at the time of recording) and the percentages of dirty and cracked eggs per cage were calculated, as well as the percentage of eggs laid into the nest. From a practical point of view, egg laying location was determined by delimiting the nest with wedges placed on the egg belt; an egg was supposed to be laid in the area in front of which it had rolled out of the cage. At 13 pm on the day previous to the measure days, all eggs blocked in the cages were gathered and the wedges were placed on the egg belt. The egg belts were stopped. At 8 am and 13 pm on the two following days, eggs were manually gathered and counted separately for the two parts of each cage.

### 2.3. Statistical Methods

During the rearing period, the experiment was designed as a 2 × 2 factorial, with beak treatment (infra-red trimmed (T) or not trimmed (NT)) and “enrichment” (enrichment (E) or no enrichment (NE)) as the two main factors, creating 4 treatments of each 3 pens with 388 birds (see Table 1).

During the laying period, the experiment was designed as a 2 × 2 × 2 factorial, with beak treatment (T or NT), enrichment (E or NE) and diet (with extra fiber cellulose (F) or control (C)) as the three main factors, creating 8 treatments of various numbers of cages (see Table 1). The treatments were systematically allocated to cages so that the eight treatments and the two levels of the three factors balanced spatially, with each tier and each battery containing each treatment (except for C treatments, which were allocated to a single battery due to practical reasons related to feed distribution).

The observational unit was the pen or the cage for ADT and NOT data and for laying data, while the single bird was considered the observational unit for body weights, feather cover data and the occurrence of beak defects and skin damage. Statistical analysis was conducted in R Studio (R 3.5.0, R Core Team, 2018, PBC, Boston, USA), separately for the rearing and laying periods.

#### 2.3.1. Single Measures

For the ratio of hens after/before NO placement during the laying period, a linear model (*lm*) was used to analyze the main and interactive effects of beak (trimmed or not), enrichment (presence or absence) and feed (with or without fiber supplementation) as fixed factors.

For NOT data (number of pullets next to the object after/before NO placement) during rearing, non-parametric testing was applied in the form of a Kruskal–Wallis test (*kruskal.test*), due to the low number of replicates. Two distinct analyses were applied with one factor: beak, enrichment.

For ex situ feather score data and body weight during laying period, the multilevel linear model (*geeglm*: generalized estimating equations) was used for variance analysis of the data. Beak, enrichment and feed were fixed effects, while cage was the random effect. Main and interactive effects were analyzed.

#### 2.3.2. Repeated Measures

For ADT data (during rearing: number of pullets within an arm’s length, touched, pecking at the observer and latency of the first peck and score during laying as ordinal data), the non-parametric testing was applied after rank transformation of data (aligned ranks transformation ANOVA: *art*) to analyze the main and interactive effects of enrichment, beak and feed during laying period as fixed factors, while cages or pens and ages were the random effects.

For hen-day egg production, percentages of dirty, cracked, and nest eggs, egg weight and for body weight data, the *geeglm* was used for variance analysis of the data. Beak, enrichment, feed and age were fixed effects, while cage or pen was the random effect. Main and interactive effects were analyzed. When interaction was significant, *post-hoc* analyses where made with Benjamini–Hochberg adjustment.

For in situ feather score data, the *geeglm* was used. Beak, enrichment and feed were fixed effects, while cage and age were the random effects. Main and interactive effects were analyzed.

For *geeglm* and *lm* analyses, the distributions of the model residuals were visually validated for normality. As the feed factor and interactive effects did not significantly affect the results, they were removed from the models.

The occurrence of mortality at the end of the rearing and laying periods, the frequency distribution of beak defects and skin damage were analyzed using a Chi-square test (*chisq.test*).

For all analyses, differences were considered significant when *p* ≤ 0.05.

### 2.4. Ethics Approval

All research was reviewed and approved by the Comité National de Réfléxion Ethique sur l’Expérimentation Animale (National committee for ethics in animal testing) No. 016 at the French Ministry for Education, Higher Education and Research before the start of data collection (number APAFIS#4703-201603251170251167 v5). All efforts were made to minimize the number of animals used and their suffering.

## 3. Results

### 3.1. Pullets

#### 3.1.1. Mortality

A total of 190 pullets died during the rearing period, none related to pecking disorders consequences. More than 80% of the mortality occurred in the first two weeks (156 out of 190). The results of a comparison between the treatments are shown in Table 2.

Mortality of T pullets was higher than that of NT pullets (6.4% vs. 1.2%, *p* < 0.001). For NT pullets only, enrichment reduced mortality: 10 pullets died in E pens (7 in the first 2 weeks), vs. 23 in NE pens, spread out in 3 pens each (9 died in the first 2 weeks).

#### 3.1.2. Body Status: Body Weight, Feather Cover, Skin Damage and Beak Defects

Between 1 and 119 days of age, body weights were not different depending on enrichment (*p* = 0.965). Beak x age interaction was significant (*p* = 0.014): NT pullets were heavier than T pullets from 3 to 21 days (after *post-hoc* adjustment), without differences thereafter.

At 16 WOA, feather cover was scored as 0 for all pullets of the sample, and no skin damage was observed. The beak was noted as having a defect for 1.9% of NT pullets and for 38.5% of T pullets (significant difference, *p* < 0.001), without a difference between E and NE pullets (22.9% vs. 16.2% respectively, *p* = 0.228).

#### 3.1.3. Novel Object Test (NOT)

The percentage of pullets approaching the NO was 24.6%, without difference between treatments (T vs. NT: *p* = 0.572, E vs. NE: *p* = 0.126). The number of pullets observed pecking at the NO was low: only 1 hen was observed pecking at the NO in 4 pens, 3 in another pen, and 0 in all the others, across the various treatment groups. This could not be subjected to further statistical analyses.

#### 3.1.4. Avoidance Distance Test (ADT)

Beak condition did not significantly affect the results of the ADTs. The number of pullets pecking at the observer tended to be higher in E pens than in NE pens (*p* = 0.060). Similarly, the latency of the first peck tended to be shorter in E pens (*p* = 0.054). Number of pullets around the observer and touched by the observer were not different between E and NE pens (*p* = 0.690 and *p* = 0.245).

### 3.2. Laying Period

#### 3.2.1. Feed Treatments

No result was significantly different between C and F hens, irrespective of the age, and interactions between feed treatment and the other main factors were never significant. The results for feed treatment comparisons are shown in Table 3, separately from enrichment and beak treatments comparisons results, given in the following sections of the paper.

#### 3.2.2. Mortality

A total of 244 hens died during the laying period from 18 to 72 WOA. Of these, 42 hens corresponded to culls, justified by pecking injuries. The results of comparisons between treatments are shown in Table 4.

Mortality of T hens was lower than NT hens: 2.55% for T hens vs. 8.89% for NT hens (*p* < 0.001, test χ^2^). For NT hens, enrichment reduced mortality; this was not the case for T hens. Mortality varied greatly between cages within the same group, especially for NT hens (0 to 26 dead hens per cage recorded over the laying period). From 31 to 35 WOA, there was one IP episode: 21 cages were affected by mortality, including 16 due to IP: 91 hens died (i.e., 37% of total mortality), particularly in six NT cages with 74 hens dying, including 32 euthanized. During the entire laying period (18 to 72 WOA), seven cages showed no mortality at all (5 were T cages, 2 were NT-E cages, none was NT-NE cages).

#### 3.2.3. Body Status: Body Weight, Feather Cover, Skin Damage and Beak Defects

The results for body status are shown in Table 5.

Trimmed hens were heavier than NT hens at 71 WOA, with a difference of 158 g, which is 8% more. The feather score was lower for T hens compared to NT hens (in and ex situ). The interaction beak treatment x enrichment was significant in situ: for NT hens, feather score was lower for E than for NE treatments (*p* = 0.002, after *post-hoc* adjustment), for T hens, enrichment had no effect on feather score. Skin damage was more frequent in NT hens than in T hens at 71 WOA. More T hens displayed beak defects than NT hens. The majority of defects were shovel beak (25% of the T hens), split beak (19%) and crossed beak (7%).

#### 3.2.4. Novel Object Test (NOT)

No hens pecked at the NO during the 2-min period of observation. The proportion of hens close to the NO (i.e., immediately behind the façade or with their head out of the façade) after NO placement in comparison to before NO placement was higher in E cages than in NE cages (22.9% vs. 6.5%, *p* < 0.001), without a difference between beak treatments (*p* = 0.390).

#### 3.2.5. Avoidance Distance Test (ADT)

No effect of enrichment (*p* = 0.214), beak (*p* = 0.162) nor interaction beak × enrichment (*p* = 0.644) on ADT was detected in this test.

#### 3.2.6. Laying Performance

Laying performances are shown in Table 6.

Laying rates, percentage of nest laying, egg weights and percentages of cracked and dirty eggs were different at different age.

Laying rate of NT hens were lower than those of T hens, with interaction with age: NT hens lay less eggs at 50, 59, 64 and 68 WOA (significant after *post-hoc* adjustment). The percentage of nest laying was higher for T compared to NT hens. Enrichment increased the percentage of eggs laid in the nest, and tended to increase the laying rate. There was no difference between treatments on egg weight, and percentages of cracked and dirty eggs.

## 4. Discussion

Supplementing feed with fiber did not result in the expected decrease of IP consequences, even when added to other management strategies. In [36,37], increase in the crude fiber content did not reduce animal losses and plumage damage neither. This is not consistent with the findings of other studies where higher crude fiber content led to better plumage condition in laying hens by reducing feather pecking and pecking damage to skin. This was explained by a prolonged feeding period, with prolonged intestinal passage, and thus a longer lasting feeling of satiety [28,38,39]. Several explanations can be made for these inconsistent results. First, fiber supplementation was kept low in our study in order to maintain high zootechnical performances for farm implementation, but possibly too low to see an effect on IP consequences. Second, interactions with other feed characteristics (energy, protein and amino acids levels) seem to be important [40]. Fibers can also be more effective if ingested through routes other than via feed, for example through foraging in the environment (hay or dehydrated alfalfa bales, [41]). Finally, if supplementation in feed with fiber had been provided as early as the pullet rearing period in our study, this could have led to reduced IP consequences. This needs further investigation.

With or without fiber supplementation in feed, the management strategies provided in our study both at the rearing and laying stages showed many positive effects on several aspects. Positive effects of management strategies were even more visible on NT animals where E treatments lead to lower pullet and laying hen mortalities, and better feather cover during the laying period. This confirms previous results observed on laying hens in floor housing systems, reviewed by [26].

In particular, birds housed in E environment were less fearful (or more attracted) towards a novel object during the laying period and to a lesser extent tended to be less fearful towards an unfamiliar human (or more attracted) during the rearing period. This suggests that E birds would have a lower risk to experience stress than NE animals, and shows the effectiveness of using multiple sources of management strategies, from the very beginning of the rearing period, from physical objects, sounds, lights and human sources. This is consistent with [42], who found, under commercial free-range conditions, significant relationships between stockperson attitudes, the number of hens that could be approached and touched, and the feather damage and mortality rates of these hens.

An interesting result was that mortality in NT pullets was rare, but even lower in E pens. The difference in mortality appeared after the first two weeks, and was not directly linked to IP related problems. This may be associated with an overall decrease in stress, and/or increase in positive emotions with secondary effects on health. In fact, enriched housing environments can have positive impacts on immunocompetence (reviewed by [26]).

Enrichment in the cage, like the artificial turf mat in the pecking and scratching area of the cage, did not relocate egg laying to this area. On the contrary, the percentage of egg laying into the nest was higher in E cages, showing better distribution of resources and a better use of the cage areas by the hens. In the same way, T hens better used the nest for egg laying than NT hens. In NE and NT cages, hens may have used the nest as an escape from peckers, making it less available for egg laying. The better use of nest for laying can partly explain the better laying rates in E (tendency) and in T cages.

The management strategies provided in the rearing up to laying stages have therefore shown their value for the welfare of animals, without a negative impact on productivity. However, this approach still showed some limitations and can be improved.

The E treatments limited feather cover damage for NT hens during the laying period, but at 71 WOA it was no longer sufficient: all hens showed severe damage of the feather cover. This shows some difficulties for field application in comparison with the beak trimming procedure. Also, during the IP episode in the laying period, NE hens were far more affected than E hens, but mortality varied greatly between cages within the same group treatment, especially for NT hens. The causes of this IP episode were unknown and probably multifactorial (it took place in the middle of the laying peak, and just after a change in feed formula). This shows the complexity of the problem and how difficult it is to control on the farm.

Applying a similar environment between the rearing and laying periods would probably play a role in decreasing the risk of IP. Our experimental setup limited the implementation of certain management strategies: one single room housed E and NE cages with the same environmental conditions for all cages. However, on commercial farms, solutions can be added during the laying period as provided during the rearing period such as music playing, warning of human arrival and improving the human-animal relationship. Altogether, this would probably further increase the effectiveness of management strategies on IP consequences reduction.

Moreover in our study, the effects of management strategies during the laying phase could be long-term effects of the rearing treatment. Indeed, in [13], where extra environmental enrichment was applied only during the laying period in furnished cages, no effect was observed on IP consequences. Whether the management strategies, applied only during the rearing period, would have had positive effects still needs to be investigated.

Our study proposed various management strategies, both at the rearing and laying stages, based on the existing literature but also on the practical and economic feasibility of installing such enrichments in commercial farms in barn system and in furnished cages. The objects provided were simple, easily obtained directly on farms or inexpensive if purchased. In barn systems, the farmer can easily renew them. However in furnished cages, we decided not to renew the objects in order to propose the simplest situation for farmers (but perhaps not as positive from the animal welfare point of view). It is thus possible that the management strategies provided in our study could be even more effective if objects were renewed during the laying period. The farmer could do this several times during the laying period, during hens’ presence as objects are placed on the façade of the cage.

Although precautions have been taken to avoid observer bias in body condition assesment, inter-observer reliability testing should be added for future studies in order to improve methodology.

Unsurprisingly, beak trimming considerably limited the consequences of IP during the laying period: lower mortality, better feather cover and lower skin damage. These results are similar to those reported in previous studies that have been conducted on commercial farms [43,44] in barn or cage housing systems. Probably as a result, T hens were heavier, likely linked to better feather cover and less energy loss for thermoregulation and recovery [45,46]. Trimmed hens also had higher laying rates than NT hens perhaps due to a lower stress level caused by IP in cages: stress can result in a decrease in egg laying rates [46].

However, negative consequences of beak-trimming were highlighted in our study. More than a third of T pullets were affected by beak defects at the end of the rearing period (reaching half at the end of the laying period). This demonstrates poor treatment of the beak and regrowth problems. This is in line with field results [6,14,47], also with infra-red beak trimming. It therefore seems that the technique still needs to be better controlled and adapted to the anatomy of the beak. Although the question of pain directly due to infra-red beak trimming or beak defects is under debate [44,48], beak defects may have led to discomfort in beak use affecting normal behavior, such as foraging, feeding, drinking, and preening [49,50]. In our study, the droppers used during rearing had an overpressure in water that could not technically be adjusted, probably making it difficult for the most sensitive individuals to drink. As a possible consequence, T birds in comparison to NT birds showed decreased body weights until 3 WOA, albeit without further decrease, as found in previous studies ([51] up to 4 WOA and [52] up to 8 WOA), and mortality was higher, especially at the beginning of rearing.

## 5. Conclusions

This study investigated practical solutions in the situation where non-beak-trimmed animals are reared in a barn system and transferred to furnished cages. Fiber supplementation in feed did not provide any added value, but different combinations of management strategies from the rearing to laying stages increased animal welfare, especially for animals with intact beaks, without negative impacts on productivity. These management strategies improved animal welfare in terms of decreased fear, mortality and improved feather cover, while remaining realistic and feasible with easy procurement of inexpensive and simple objects on the farm.

As beak trimming is increasingly coming under scrutiny, such management strategies should be reinforced on farms even before any ban on beak trimming. This process should involve pullet flocks, in addition to laying hen flocks, as they are often not included in the debates around IP. Although these enrichment solutions can be improved, they achieved their goal: decreasing IP consequences when hens were not beak-trimmed. However, this study also shows the complexity of resolving the multifactorial IP problem entirely. Despite improved housing and flock management, the risk of IP cannot be completely eliminated. If still used, the infra-red beak trimming technique needs to be better controlled.

## Figures and Tables

**Table 1:** Number of pens of 388 pullets during the rearing period and cages of 60 hens during the laying period per treatment.

Beak	Enrichment	Rearing Period	Laying Period	
		Normal Diet	F Diet	C Diet
**T**	**E**	3 pens	12 cages	5 cages
	**NE**	3 pens	11 cages	6 cages
**NT**	**E**	3 pens	12 cages	6 cages
	**NE**	3 pens	12 cages	6 cages

T: beak-trimmed, NT: not beak-trimmed, E: with enrichment, NE: without enrichment. F: diet with extra fiber cellulose, C: control diet.

**Table 2:** Mortality at 17 weeks of age (WOA) (% of chicks placed at day 1 of age).

Beak Treatment	E	NE	*p* Enrichment ^1^
**T**	6.96%	6.53%	0.679
**NT**	0.86%	1.98%	0.022
***p* beak ^2^**	<0.001	<0.001	

T: beak-trimmed, NT: not beak-trimmed, E: with enrichment, NE: without enrichment. ^1^ *p* indicates the significance rate of the χ^2^ test for E/NE treatment comparisons; ^2^ *p* indicates the significance rate of the χ^2^ test for T/NT treatment comparisons.

**Table 3:** Mortality, body status, NOT, ADT and laying performances results. For repeated measures, data are shown as averages. n body status = 8 hens × 47 F and 23 C cages, n NOT and ADT = 31 F and 15 C cages, n laying performances = 26 F and 23 C cages.

Measures		Age (Weeks)	F	C	*p*-Value
Mortality (%)		72	6.13	5.14	0.197 ^1^
Body status	Feather score (*in situ*)	4 controls from 31 to 61 WOA	1.60	1.54	0.824
	Feather score (*ex situ*)	71	3.41	3.62	0.275
	Weight (g)	71	2076	2110	0.069
	Skin damage (% hens observed)	71	12.88	11.97	0.750 ^1^
	Beak defect (% hens observed)	71	26.07	29.49	0.370 ^1^
NOT	Hens close to the NO (%)	62	14.1	16.0	0.552
ADT	Score	3 tests: 31, 41, 53 WOA	2.87	2.77	0.280
Laying performances	Laying rate (%)	8 controls from 24 to 72 WOA	88.7	89.4	0.593
	Egg weight (g)		62.5	62.5	0.933
	Dirty eggs (%)		2.6	2.3	0.415
	Cracked eggs (%)		0.6	0.6	0.515
	Eggs in nest (%)		97.2	97.4	0.619

F: diet with extra fiber cellulose, C: control diet. NOT: novel object test, ADT: avoidance distance test. *p* indicates the significance rate of the feed factor in the models [Beak + Enrichment + Feed + interactions] + age in case of repeated measures, except where ^1^ *p* indicates the significance rate of the χ^2^ test for F/C treatments comparisons.

**Table 4:** Mortality at 72 WOA (% of hens placed in cages at 18 WOA).

Beak Treatment	E	NE	*p* Enrichment ^1^
**T**	2.84%	2.25%	0.399
**NT**	6.30%	11.48%	<0.001
***p* beak ^2^**	<0.001	<0.001	

T: beak-trimmed, NT: not beak-trimmed, E: with enrichment, NE: without enrichment. ^1^ *p* indicates the significance rate of the χ^2^ test for E/NE treatment comparisons, ^2^ *p* indicates the significance rate of the χ^2^ test for T/NT treatment comparisons.

**Table 5:** Body weight (g), feather cover score and percentages of hens with skin damage and beak defect. Feather score 0: no damage, 6: very degraded feather cover. *n* = 8 hens × 17 to 18 cages per treatment.

Measures	Age (Weeks)	Beak		Enrichment		*p*-Value		
		T	NT	E	NE	Beak	Enrichment	Beak × Enrichment
Weight	71	2169	2011	2096	2085	<0.001	0.704	0.052
Feather score in situ	31 to 61	0.59	2.57	1.35	1.82	<0.001	0.009	0.007
Feather score ex situ	71	2.54	4.47	3.52	3.48	<0.001	0.674	0.986
Skin damage	71	6.01	19.13	11.07	13.93	<0.001	0.307	-
Beak defect	71	50.18	4.33	29.29	25.71	<0.001	0.344	-

T: beak-trimmed, NT: not beak-trimmed, E: with enrichment, NE: without enrichment.

**Table 6:** Laying rate (hen-day egg production), percentage of eggs laid into the nest, dirty and cracked eggs and egg weight (g), means of 8 recordings between 24 to 72 WOA, *n* = 12 to 13 cages per treatment.

Measure	Beak		Enrichment		*p-*Value					
	T	NT	E	NE	Beak	Enrichment	Age	Age × Beak	Age × Enrichment	Beak × Enrichment
Laying rate	91.0	87.1	89.8	88.3	<0.001	0.066	<0.001	<0.001	0.531	0.088
Eggs in nest	97.9	96.6	97.6	96.9	<0.001	0.005	<0.001	0.929	0.402	0.496
Egg weight	62.3	62.7	62.5	62.5	0.370	0.910	<0.001	0.120	0.801	0.560
Dirty eggs	2.6	2.3	2.6	2.3	0.194	0.171	<0.001	0.307	0.540	0.106
Cracked eggs	0.5	0.6	0.5	0.6	0.111	0.408	<0.001	0.308	0.586	0.357

T: beak-trimmed, NT: not beak-trimmed, E: with enrichment, NE: without enrichment.
